# Determinants of complete immunization among senegalese children aged 12–23 months: evidence from the demographic and health survey

**DOI:** 10.1186/s12889-017-4493-3

**Published:** 2017-07-06

**Authors:** Mouhamed Abdou Salam Mbengue, Moussa Sarr, Adama Faye, Ousseynou Badiane, Fatou Bintou Niang Camara, Souleymane Mboup, Tandakha Ndiaye Dieye

**Affiliations:** 1 IRESSEF: Institut de Recherche en Santé, de Surveillance Epidemiologique et de Formations-Dakar, Arrondissement 4 Rue 2 D1. Pole urbain de Diamniadio, 7325 Dakar, BP Senegal; 2 University of the Witwatersrand, Faculty of Health Sciences. School of Public Health, Johannesburg, South Africa; 3 Westat, Rockville, Maryland USA; 4 Department of Public Health and Preventive Medicine Cheikh Anta Diop University- Dakar, Dakar, Senegal; 5 Division of Immunization / Expanded Program on Immunization, Ministry of Health-Dakar, Dakar, Senegal; 6 Agence National de la Statistique et de la Démographie-Dakar, Dakar, Senegal; 7 Laboratory of Immunology, Cheikh Anta Diop University- Dakar, Dakar, Senegal

**Keywords:** Childhood, Immunization, Coverage, Vaccines, EPI, Senegal

## Abstract

**Background:**

The expanded Programme on Immunization (EPI) is one of the most cost-effective interventions to reduce childhood mortality and morbidity. However, determinants of childhood immunization have not been well studied in Senegal. Thus, the aim of our study is to assess routine immunization uptake and factors associated with full immunization status among Senegalese children aged 12–23 months.

**Methods:**

We used the 2010–2011 Senegalese Demographic and Health Survey data. The DHS was a two stages cross-sectional survey carried out in 2010–2011. The analysis included 2199 children aged 12–23 months. The interviewers collected information on vaccine uptake based on information from vaccination cards or maternal recall Univariate and multivariable logistic regressions models were used to identify the determinants of full childhood immunization.

**Results:**

The prevalence of complete immunization coverage among boys and girls based on both vaccination card information and mothers’ recall was 62.8%. The immunization coverage as documented on vaccination cards was 37.5%. Specific coverage for the single dose of BCG at birth, the third dose of polio vaccine, the third dose of pentavalent vaccine and the first dose of measles vaccine were 94.7%, 72.7%, 82.6%, and 82.1%, respectively. We found that mothers who could show a vaccination card [AOR 7.27 95% CI (5.50–9.60)], attended at least secondary education level [AOR 1.8 95% CI (1.20–2.48)], attended four antenatal visits [AOR 3.10 95% CI (1.69–5.63)], or delivered at a health facility [AOR 1.27 95% CI (1–1.74)] were the predictors of full childhood immunization. Additionally, children living in the eastern administrative regions of the country were less likely to be fully vaccinated [AOR 0.62 95% CI (0.39–0.97)].

**Conclusions:**

We found that the full immunization coverage among children aged between 12 and 23 months was below the national (> 80%) and international targets (90%). Geographic area, mother’s characteristics, antenatal care and access to health care services were associated with full immunization. These findings highlight the need for innovative strategies based on a holistic approach to overcome the barriers to childhood immunization in Senegal.

## Background

Childhood immunization still remains one of the most cost-effective preventive strategies against mortality and morbidity among children [1]. Immunization saves the lives of up to three million children’s every year. High rates of vaccine coverage could prevent an additional 1.6 million deaths a year among children under the age of five [1, 2].

Following WHO efforts to eradicate smallpox and control childhood diseases, Senegal launched its Expanded Programme on Immunization (EPI) in 1979. The goal of the national EPI is to ensure full immunization of children against preventable diseases. EPI is a routine activity within the public healthcare system. In addition, time specific mass immunization campaigns and door-to-door activities are regularly implemented across the country to help increase routine immunization uptake.

At the beginning of the program in 1979, the national EPI targeted seven diseases. The number of diseases covered later on increased to 9 in 2002, and then to 11 in 2005. The current immunization schedule for children 0 to 15 months old is shown in Table 1.

**Table 1:** Vaccine, year of introduction, and recommended immunization schedule in Senegal

Vaccine	Year	Doses	Age of administration
BCG	1979	1	Soon after birth
Polio 0^a^	1988	1	
			OPVo: Soon after birth
IPV	2015	3	IPV1: at 6 weeks IPV2: at 10 weeks IPV3: at 14 weeks
Hepatitis B	2016	1	Within 24 h after birth
Pentavalent vaccine			Penta 1: at 6 weeks
	2005	3	Penta 2: at 10 weeks
			Penta 3: at 14 weeks
PCV	2013	3	Pneumo 1: at 6 weeks
			Pneumo2: at 10 weeks
			Pneumo 3: at 14 weeks
Measles and Rubella	2014	1	9 months
Rotavirus	2014	2	Rota1: at 6 weeks
			Rota2: at 10 weeks
Yellow fever	1988	1	At 9 months
Measles and Rubella	2014	1	Second dose at 15 months

*BCG* Bacillus Calmette-Guerin

*Penta* diphtheria, tetanus, pertussis, haemophilus influenzae b and hepatitis B vaccine

*OPV* Oral Polio Vaccine

*IPV* Inactivated Polio Vaccine

*PCV* Pneumococcus conjugated vaccine

*N* weighted total number of children

^a^Polio 0 is the oral polio vaccination given at birth

In Senegal, EPI is funded by the government and its partners (GAVI, UNICEF, and WHO) and vaccines are provided free of charge in public health facilities. Overall, the total cost of the routine immunization program in 2013 was $17,162,250 (USD) [3]. However, despite the large sums spent to purchase vaccine the country is still struggling to maintain a steady and high rate of immunization coverage. In 2010, the reported global immunization coverage dropped down to 70%, with some districts reporting rates ≤50% for some specific vaccines. Additionally, in 2012 up to 14,940 infants did not even receive a single dose of BCG vaccine while many areas faced low use of immunization services with a DTP3 coverage between 50 and 79% [4]. The country experienced within the last 5 years a rise in measles outbreaks with high case fatality rate [5].

The breakdowns of the program are probably due to the fact that childhood immunization is a complex process involving health services organization, cultural beliefs, parent’s characteristics and socio-economic factors [6]. Beyond the financial investment, national EPI programs should integrate in a coherent approach all the modifiable factors that can affect the immunization process. Moreover, implementing a sustainable immunization program requires also a reliable cold chain and transport systems, with adequate training of health workers and appropriate educational programs to inform the communities, mothers and caregivers about available services [6].

In Senegal, most of the studies on childhood immunization focused on specific areas or on a small number of vaccine-preventable diseases [5, 7, 8]. In addition, these studies mainly studied individual characteristics rather than determinants related to access to health care and environmental factors. Finally, existing research did not sufficiently use nationally representative household surveys to assess full immunization coverage and its determinants in the country.

Thus, we conducted a secondary analysis of the 2010–2011 Demographic and Health Survey (DHS) to assess routine immunization uptake and factors associated with full immunization among children aged between 12 and 23 months. A better understanding of these determinants might help the national EPI program to re-orient its approaches with new strategies that may further reduce mortality and morbidity among children in Senegal.

## Methods

### Study design and study settings

We conducted a secondary analysis of the Senegal 2010–2011 DHS data. The objectives,organization, and sample design of the DHS are described elsewhere [9]. Briefly, the 2010–2011 DHS was a nationally representative household survey implemented across all the 14 administrative regions of Senegal between October 2010 and April 2011.

### Participants

During the 2010–2011 DHS, a total of 392 clusters were selected from urban and rural strata with a sampling probability proportional to the population size, with 147 clusters selected for urban areas and 245 clusters for rural areas. For the urban strata, the number of clusters per region varied from a maximum of 30 in Dakar to a minimum of 6 in the Kaffrine region. For the rural stratum, the number of clusters ranged from 4 in the Dakar region to 23 in the Diourbel region. At the second stage of the sampling, interviewers randomly sampled households within each cluster. A total of 8212 households were selected across the 14 regions in Senegal.

All women aged between 15 and 49 years old who were either a permanent resident or a visitor present of the household on the night before the survey were eligible for the surveys. However, the overall sample size was only representative of the national population and couldn’t be disaggregated by region. Our analysis included women who had a live birth within the 2 years preceding the survey and with a living child aged 12–23 months.

### Survey instrument and administration

Eligible women were interviewed using a Women’s Questionnaire, including maternal and child parameters [10]. The 2011 DHS collected information on vaccination coverage from two sources: the vaccination card shown by mothers to interviewers and the mother’s recall of vaccination. If the health card was available, information regarding the date of administration was directly collected from the vaccination card which normally records dates of all routine vaccinations. If no card was presented, the interviewer asked the mother to recall all vaccination received by their child and when appropriate, the number of doses received without asking for the dates. During the 2011 DHS, out of a total of 2199 women surveyed, 31.33% (689/2199) did not show a vaccination card and therefore reported on children vaccination by recall only [10]. Evidence on the quality of data from maternal recall is documented from previous studies in sub-Saharan Africa and LMICs which indicate that maternal recall is almost similar compared to data collected from the health card [11, 12].

### Measurement of variables

#### Outcome variable

We used the Children’s record dataset for those aged between 12 and 23 months. According to WHO guidelines, a fully immunized child is a child in the 12–23 months old age group who has received a single dose of BCG vaccine, three doses of Pentavalent vaccine (which contains five antigens against diphtheria, tetanus, pertussis, hepatitis B and haemophilus type b), three doses of polio vaccine (excluding the dose given shortly after birth) and the first dose of measles vaccine. In this study, our definition of full immunization did not include vaccines introduced after 2012 such as rotavirus vaccine, pneumococcal vaccine and the second dose of measles-contained vaccine. Previous studies on child immunization coverage have used the same definition for full childhood immunization [13–15]. Thus, the percentage of children aged between 12 and 23 months who received all the specified vaccines according to the information on vaccination card or by mother’s recall represents the study outcome.

In the children’s dataset, the outcome “complete immunization” had five categories of response: No (o), vaccinations dates on card (1), vaccinations reported by mothers (2), vaccinations marked on card (3) and don’t know (4). We recoded the three categories “vaccination date on card” (1), “vaccination reported by mothers” (2) and “vaccination marked on card” (3) as “1” and recoded all the remaining categories as “0” and labelled “not received”. For the nine vaccine doses, we first recoded to reflect “vaccinated” or “not vaccinated” for each dose and combined them to reflect “completely vaccinated”.

#### Exposure variables

We selected 17 co-factor variables potentially associated with child immunization. The WHO framework on epidemiology of the unimmunized child [6] describes the different factors affecting child’s immunization into four main categories: health care immunization system, communication and information, family characteristics, and parental attitudes and knowledge. In our study, the immunization system category included the distance to health facility, and the need to take transportation. The communication and information category included: use of mass media according to the levels of access and source (radio, TV and newspapers), family characteristics included the followings variables: mother’s and father’s education level, mother’s age at child birth, marital status, household level of poverty assessed by the wealth quintile, ethnic group, religion, child gender, birth order, urban/rural residence (urban/rural), and region of residence. Variables on familiarity and use of other health care services such as antenatal care during pregnancy and the relative distance to the closest health center represented the parental attitudes/knowledge. Finally, we included the gender relationship such as the involvement of women in household decision making.

### Statistical analysis

We summarized continuous variables using means with standard deviations and summarized categorical variables with frequencies and percentages. We conducted bivariate analysis and binomial logistic regression. Variables significant at *p*-value ≤0.25 were included in the multivariable logistic regression models. Vvariables that did not have a significant regression coefficient were removed and a smaller model set up. To assess for confounding, we compared for each variable the estimated coefficient in the smaller model with the previous values in the larger model. Variables, when excluded, that changed the coefficient of remaining variables of Δβ > 20%, were considered as potential confounders and were added back in the model [16]. Variables that were not significant at the univariate analysis were added back to the model and their significance assessed in the presence of other significant variables. Finally, we added demographic characteristics such as age, sex, and predictors well known from previous research but not significant in our model. Subsequently, the goodness of fit of our final model was tested using the Hosmer-Lemeshow test [17]. All data management procedures and statistical analysis were done using STATA software version 13. Due to the complex sampling design, we used the Svyset command to account for inverse probability weighting (IPW), clustering, and stratification to provide unbiased estimates of the population parameters.

## Results

### Sociodemographic characteristics of children and mothers

As indicated in Table 2, the mean age (±SD) of mothers in the sample was 27.5 years (±2.23). More than half of women (68.7%) had no formal education and nearly 50% (43.8%) were classified as “poor/poorest” (Table 2). A total of 2199 children aged between 12 and 23 months at the time of the interviews were included in this study. Just over half (51.27%) of the children in the sample were males and 31.36% of them did not have a vaccination card.

**Table 2:** Characteristics of the study population

Baseline characteristics	Number of respondents	Proportion %
Mother’s age at birth		
Mean (± SD)	27.5 (±2.23)	
<20	310	14.4%
20–34	1527	69.6%
35–49	346	16.0%
Mother’s education level		
No education	1510	68.7%
Primary	515	23.4%
Secondary	168	7.7%
Higher	6	0.3%
Religion		
Muslim	2137	97.2%
Christian	52	2.4%
Animist	8	0.4%
No religion	1	0.03%
Other	1	0.01%
Marital status		
Never in union	82	3.7%
Married	2044	93.0%
Living with partner	17	0.7%
Widowed	12	0.5%
Divorced	35	1.6%
No longer living together/separated	11	0.5%
Mother’s ethnic group		
Wolof	823	37.4%
Puular	637	29.0%
Serer	313	14.2%
Manding	97	4.4%
Diola	75	3.4%
Soninke	53	2.4%
Not a Senegalese	59	2.7%
Other	141	6.5%
Wealth quintile		
Poorest	493	22.4%
Poor	470	21.4%
Middle	452	20.6%
Richer	472	21.5%
Richest	312	14.2%
Place of residence		
Urban	850	39.0%
Rural	1349	61%

### Vaccination coverage rate among children aged between 12 and 23 months

The prevalence of complete immunization coverage among all children was 62.8% (Table 3). However, the prevalence of complete immunization coverage obtained by information from the vaccination card was 37.5%, while the same information obtained by mother’s recall of vaccination was 77.14%.

**Table 3:** Immunization coverage by region among children aged 12–23 months during the DHS, 2010–2011

Vaccines	National *n* = 2199	West *n* = 1534	North *n* = 246	East *n* = 136	South *n* = 283
BCG	2083 (94.70%)	1467 (95.63%)	228.6 (93.10%)	266 (94.00%)	119 (88.40%)
Polio 0^a^	1741 (79.20%)	1291 (84.13%)	180.4 (73.40%)	77 (57.43%)	191 (67.64%)
Penta1	2063 (93.80%)	1450 (94.45%)	224 (91.05%)	122 (89.00%)	279 (98.50%)
Penta 2	1998 (90.80%)	1414 (92.17%)	216 (87.80%)	114 (70.00%)	257 (90.81%)
Penta 3	1817 (82.60%)	1301 (84.81%)	188 (76.42%)	95 (69.80%)	238 (84.10%)
0PV1	2081 (94.60%)	1462 (95.30%)	225 (91.40%)	123 (90.40%)	272 (96.11%)
0PV2	1994 (90.70%)	1412 (92.04%)	212 (86.50%)	115 (84.60%)	257 (90.81%)
0PV3	1598 (72.70%)	1133 (73.85%)	169 (68.70%)	82 (60.74%)	218 (77.00%)
Measles	1805 (82.10%)	1133 (73.85%)	134 (54.50%)	62 (46.00%)	194 (66.87%)
All antigens^b^	1381 (62.80%)	996 (64.90%)	187 (76.01%)	101 (74.20%)	235 (83.30%)

*BCG* Bacillus Calmette-Guerin

*Penta* diphtheria, tetanus, pertussis, hepatitis B vaccine and haemophilus influenzae b

*0PV* Oral Polio Vaccine

*PCV* Pneumococcus conjugated vaccine

*N* weighted total number of children

^a^Polio 0 is the oral polio vaccination given within 24 h after birth

^b^Includes a single dose of BCG vaccine, three doses of Pentavalent vaccines and three doses of Polio vaccine (excluding the birth) and the first dose of measles vaccine

For specific vaccines, the immunization coverage for antigens given at birth were 94.7% and 79.2% for BCG vaccine and for the oral polio vaccine, respectively (Table 3). For vaccines given at 6 weeks of age, the coverages were 93.8% for Penta1 was 93.8% and 94.6% for Polio1. Finally, for vaccines provided at 10 months, 14 months, and 15 months, the immunization coverages were 82.6% for Penta 3, 72.7% for Polio 3, and 82.1% for Measles (Table 3).

For geographic differences, the southern regions of Senegal had the highest percentage of children fully immunized with a coverage of 66.87% while the lowest percentage was seen in the eastern side of the country with a coverage of children fully immunized at 46% found in the eastern administrative regions (Table 3). Finally, for economic differences, the percentage of fully immunized children was the highest among the “richest” families at 70.7% (Table 3). That same percentage dropped down to 63.8% for the “mid-level of wealth” families and was the lowest at 56.6% for the “poorest” families.

### Determinants of immunization status among children aged between 12 and 23 months

Tables 4 and 5 show the results of the bivariate and multivariate analysis to identify factors associated with full immunization status.

**Table 4:** Immunization status by parental and child characteristics in Senegal (2010–2011 DHS)

Variables	Number of children	Fully immunized n (%)	*p-*value
Mother’s age at birth			
< 20	319	198 (62.2%)	
20–34	1527	967 (63.4%)	0.860
35–49	353	216 (61.1%)	
Mother’s education level			
No education	1510	918 (60.8%)	
Up to primary	515	331 (64.4%)	0.002
Secondary and higher	174	132 (76.1%)	
Marital status			
Married or living with partner	2060	1280 (62.2%)	
Others	139	73 (101.8%)	0.024*
Father’s education			
No education	1695	1040 (61.4%)	
Up to primary	217	129 (59.3%)	0.067
Secondary and higher	205	147 (72.0%)	
Father’s occupation			
Not working	57	29 (50.8%)	
Management/Professional/Clerical/sale service	839	537 (64.0%)	0.151
Agriculture	553	321 (58.0%)	
Manual or household worker/other	572	363 (64.0%)	
Mother’s ethnic group			
Wolof	823	537 (65.3%)	
Puular	637	348 (54.6%)	0.005*
Serer	720	496 (67.2%)	
Region			
West	1534	997 (64.9%)	
North	245	133 (54.5%)	0.001*
East	135	189 (66.8%)	
South	282	62 (46.0%)	
“Mother could show a vaccination” card?			
Yes	689	218 (31.6%)	< 0.001*
No	1510	1164 (77.1%)	
Wealth quintile			
Poorest	485	274 (56.4%)	
Poor	457	283 (61.9%)	
Middle	446	284 (63.8%)	0.061
Richer	466	296 (63.6%)	
Richest	307	217 (70.7%)	
Place of residence			
Urban	850	535 (63.1%)	
Rural	1349	844 (62.7%)	0.850
Sex of the child			
Male	1127	708 (62.8%)	
Female	1072	673 (62.8%)	0.955
Birth order			
0–3	128	778 (60.7%)	
4–6	368	229 (62.3%)	0.324
≥ 7	66	48 (73.0%)	
Read newspaper			
Not at all	1886	1175 (62.3%)	0.440
Less than once per week or at least once a week	276	180 (65.2%)	
Listen to radio			
Not at all	379	230 (60.5%)	0.403
Less than 1 week or at least 1 week	1782	1125 (63.1%)	
Watch television			
Not at all	734	421 (57.4%)	0.003*
Less than 1 week or at least 1 week	1427	933 (65.4%)	
Distance to health care Center			
As a big problem	777	465 (59.9%)	0.646
As not a big problem	1385	889 (64.2%)	
Religion			
Muslim	2101	1316 (62.6%)	0.771
Others	60	39 (64.7%)	
Use of antenatal care			
Yes	85	1254 (64.4%)	< 0.001*
No	1948	27 (32.2%)	
Place of delivery			
At hospital	1616	1069 (66.2%)	< 0.001*
At home	583	312 (53.5%)	
Woman participates in household decisions			
Yes	530	335 (63.2%)	0.216
No	1492	918 (61.5%)	

Data are expressed as means (SDs) or percentages.

*Denotes *P*-value statistically significant at 0.05 level

**Table 5:** Factors associated with full immunization of children 12–23 months in Senegal (2010–2011 DHS)

Variables	OR (95% CI)	AOR (95% CI)	*P* value
Mother’s age at birth			
< 20	1	1	
20–34	1.06 (1.06–0.14)	1.13 (0.80–1.53)	0.396
35–49	1.01 (0.70–1.45)	0.8 (0.60–1.34)	0.575
Child sex			
Male	1	1	
Female	0.99 (0.81–1.21)	0.99 (0.80–1.21)	0.875
Mother’s education level			
No education	1	1	
Up to primary	1.16 (0.89–1.51)	1.04 (0.78–1.30)	0.728
Secondary and higher	2.2 (1.41–3.41)	1.81*(1.20–2.48)	0.005*
Ethnic group			
Wolof	1	1	
Puular	0.70 (0.50–0.86)	0.81 (0.60–1.14)	0.250
Serer and others	1.12 (0.87–1.45)	1.13 (0.85–1.51)	0.382
Access to information			
Yes	1.21 (0.90–1.60)	1.10 (0.80–1.50)	0.588
No	1	1	
Place of delivery			
At health facility	1.68 (1.31–2.12)	1.67*(1.31–2.12)	0.049 *
At home	1	1	
ANC visit during pregnancy			
Yes	3.80 (2.33–6.21)	3.10*(1.69–5.63)	< 0.001 *
No	1	1	
Distance to health facility not a problem			
Yes	1.20 (0.96–1.50)	1.03 (0.78–1.35)	0.808
No	1	1	
Area of residence			
Urban	0.90 (0.80–1.26)	1.20 (0.85–1.71)	0.093
Rural	1	1	
Region			
West	1	1	
North	0.65 (0.43–1.00)	0.81 (0.53–1.24)	0.343
South	1.08 (0.30–0.55)	1.62 (0.98–2.08)	0.059
East	0.47 (0.79–1.18)	0.62 (0.39–0.97)	0.039*
“Mother could show a vaccination” card?			
Yes	7.35 (5.18–8.60)	7.27*(5.50–9.60)	< 0.001*
No	1	1	
Wealth index			
Poorest	1	1	
Poor	1.20 (0.90–1.60)	1.15 (0.84–1.58)	0.986
Middle	1.40 (0.90–1.80)	1.04 (0.69–1.56)	0.572
Rich	1.40 (0.97–1.90)	1.31 (0.80–2.15)	0.179
Richest	1.80 (1.20–2.96)	1.45 (0.88–2.74)	0.042

*OR* Odds Ratio

*AOR* Adjusted Odds Ratio

*CI* Confidence Interval

Data are expressed as means (SDs) or percentages

*Denotes *P* value statistically significant at 0.05 level

For the bivariate analysis, factors associated with full immunization coverage were mother’s education level, mother’s marital status, mother being able to show a vaccination card, access to information from television and place of delivery (Table 4). The prevalence of fully immunized children was not significantly different between urban and rural areas (63.1% vs 62.7% *p* value = 0.85).

Results of multivariable logistic regression analysis (Table 5) show that children aged between 12 and 23 months whose mother attended at least secondary school were more likely to be fully vaccinated (AOR 1.8 95% CI [1.20–2.48] *p* value = 0.001) compared to children with lower educated parents (Table 4). In addition, Table 5 indicates that women with four antenatal visits during pregnancy were more likely to have their children vaccinated by the age of 12–23 months than those with lower number of antenatal visits [AOR 3.10 95% CI (1.69–5.63) *p* value <0.001]. Similarly, children whose mothers delivered in health facilities were more likely to be vaccinated than those whose mothers delivered at home [AOR 1.27 95% CI (1–1.74) *p* value = 0.049] (Table 5).

Table 5 shows that children whose mothers were able to show a vaccination card were 7.27 times more likely to be fully vaccinated as compared to children whose mothers could not show a vaccination card during the survey [AOR 7.27 95% CI (5.50–9.60) *p* value <0.001]. Likewise, children living in eastern regions were less likely to be fully vaccinated as compared to children living in western regions [AOR 0.62 95% CI (0.39–0.97) *p* value = 0.039] (Table 5).

Variables such as mother’s age, household wealth quintile, and “urban versus rural areas” were not independently associated with full immunization coverage.

## Discussion

We assessed routine immunization uptake and factors associated with full immunization among 12–23 months old children in Senegal. Our results show that among these children more than half of them received the nine recommended vaccines and the overall immunization coverage was 62.8%. Based on mother’s recall, 77.14% of children were vaccinated while only 37.5% were fully immunized based on information from the vaccination card. We found that children aged between 12 and 23 months whose mother showed a vaccination card, attended at least a secondary education level, attended antenatal care, or delivered at a health facility were more likely to be fully immunized. Moreover, children living in eastern regions were less likely to be immunized or complete their vaccination schedule when compared to children living in western regions.

The coverage of 62.8% felt short below the global immunization goal and strategy (GIVS) recommended target of ≥90% national immunization coverage set by WHO and UNICEF. However, this prevalence is still high when compared to what is seen in other West African countries such as Guinea: 30% [3], Ivory Cost: 51% and Mali: 39% [18]. The variations in immunization coverage between different countries can be explained by factors such as socio-cultural, or health services coverage and performance differences [6].

In this study, the proportion of children who receiving the BCG vaccine (95%) was higher than those receiving the first dose of the polio vaccine (79%). This finding indicates there are still missed opportunities and highlight the challenge of introducing early polio vaccine which should be given within 24 h after birth.

In our study, there was a decline in coverage of immunization from BCG at birth (94.7%) to measles (82.1%). Overall, the dropout rate between BCG vaccine and measles vaccine was around 11%.

Our findings corroborate with those in Nigeria, Guinea, and Uganda [18–20]. A plausible reason to explain reduction in the proportion of full vaccination coverage when children get older compared to vaccines received after birth may be due to logistical problems but also the fact that some mothers may not understand the routine immunization schedule [6] or may not choose to come back after adverse events following the first contact with the immunization system. In a study in West Africa, factors determining completion of the DTP3/Oral polio vaccine included past experience with vaccination services (short waiting time, not having been turned away or not knowing a child with post vaccine adverse events). The high dropout rate means that difficulties still exist in immunization program utilization specifically the follow-up of children throughout the immunization schedule [6].

Our study revealed that 77.14% of children were fully immunized according to mothers recall while only 37.5% were classified as fully immunized when only the vaccination card was considered. Similar findings in the differences between the two methods have been previously described in Nigeria and Ethiopia [13, 20]. In addition to recall bias, this difference may be the result of mothers without a health card to tend to provide answers that will be viewed favorably by the interviewers, or social desirability bias. Thus, this situation may lead to overestimation of doses really received by the children.

Our results indicate that out of the four main geographic areas, the eastern regions had the lowest rate of immunization coverage and there is significant regional difference as children from southern regions had the highest rates. This difference may be due to differences in the uptake of immunization services based on cultural beliefs or differences in the quantity and/or the quality of health care services between the administrative regions [7, 21]. This may also be explained by, vaccine procurement, supply, cold-chain, or differences in other logistics issues between regions [22, 23].

In this study, children born from mothers who attended antenatal care during pregnancy or who gave birth at the health facility were more likely to be fully vaccinated. Similar findings were seen on other studies in LMICs and sub-Saharan African countries [6, 13–15]. This may be explained by the fact that in Senegal local health centers are the backbone of the primary health care approach and offer a range of preventive and curative services including immunization programs, which have been strengthened at the district level between 2001 and 2005 following WHO recommendations. An intervention called “The Reaching Every District strategy” [15, 22, 24] has been used to strengthened the immunization system and support low performance health districts. With the adoption of this strategy, public health facility managers were encouraged to implement the principle of good immunization practices. This strategy also included the identification, and resolution of local problems, organization of regular outreach in vaccine delivery services and community participation in raising vaccine coverage. Another strategy adopted at the district level in Senegal was the integration of childhood immunization into all aspects of child health care delivery.

The capacity to achieve equity is a key component of national immunization programmes [21]. In this study, we analyzed equity in immunization coverage with regard to areas of residence (urban versus rural) and the household wealth index (full immunization coverage by wealth quintile). Our multivariate analyses results showed no significant associations between full immunization and areas of residence or household wealth index. These findings were not confirmed by results seen in other African and other LMI countries [4, 12, 13, 16, 18, 25]. In a study in Ethiopia, Lakew et al. [13] showed that children born from mothers of higher wealth index were 40% more likely to have received full vaccination status compared with children from women of poor wealth index group. However, in their study, the wealth quintile was categorized only into three groups (poor, middle and rich) and the percent coverage among children in the lowest quintile group was very low compared to our study (17.5% vs 61.9%). Similarly, Lynch et al. demonstrated that higher economic status was associated with better health [25]. However, in their study they used a more complex definition of health including quality of life, life expectancy, and specific measure such as causes of death [25].

Our study focused on nine vaccines available at the time of the survey used for theses analyses. However, in 2013 and 2014, Senegal launched a large-scale introduction of the pneumococcal vaccine and the rotavirus vaccines. Additionally, a human papilloma virus (HPV) vaccine to prevent cervical cancer among adolescent girls aged 9–13 years old is expected to be added to the vaccines offered in 2017. With the introduction of these new antigens into the routine immunization schedule, the number of vaccines offered by the national EPI through the public health infrastructures is expected to rise. However, there is a risk that the introduction of these new vaccines may negatively impact the delivery of traditional vaccines such as BCG vaccine, pentavalent, measles and polio vaccine [26]. Therefore, current strategies should focus on planning and strengthening the health system to make sure the rates of immunization coverage is not affected by the addition of new vaccines.

## Limitations of the study

Our study has some limitations. First, information on child’s immunization was collected from either the health card or the mother’s recall of vaccinations as recommended by the World Health Organization. However, because of potential shame and social stigma, mothers of children who don’t have the vaccination recorded on the health card may be more tempted to report a vaccination for their children introducing a potential subject bias. Consequently, the level of immunization coverage may be lower than the prevalence reported in this study. Second, information on immunization and certain sociodemographic characteristics was collected at the same time, therefore it may be difficult to establish a causal relationship between these characteristics and the child immunization status. Third, in this study, based on the birth history, only living children were included, therefore the generalizability to all children, living and deceased, remains unclear.

## Conclusions

In conclusion, we found that immunization coverage among children aged between 12 and 23 months was below the national (> 80%) and international objectives (> 90%). Our findings show that childhood full immunization depends on different factors related to mother’s individual characteristics, household characteristics and the use of antenatal services. These findings highlight the importance of using data to target specific geographic areas or age groups as well as address several factors that contribute to lower immunization rates.

## Abbreviations

ANSD: Agence Nationale de la Statistique et de la Démographie
AOR: Adjusted odds ratio
BCG: Bacillus of Calmette Guerin
CI: Confidence interval
DHS: Demographic and Health Survey
DPT: Diphteria, Pertussis Tetanus
EPI: Expanded Program on Immunization
GAVI: Global Alliance Vaccine Initiative
GIIV: Global Initiative for Independence in Vaccine
GIVS: Global immunization vision strategy
Hib: Haemophilus influenzae type B
HPV: Human papilloma virus
IEC: Information Education and Communication
IPV: Inactivated polio vaccine
IQR: Interquartile range
LMICs: Low and middle-income countries
MOH: Ministry of Health
MR: Muss-rubella vaccine
OPVo: Oral Polio Vaccine at birth
OR: Odds ratio
Penta: Pentavalent vaccine
PSUs: Population sampling units
RED: Reaching Every District
Rota: Rotavirus
SD: Standard deviation
TV: Television
UNICEF: United Nation Children’s Fund
USD: US dollar
VII: Vaccine Independent Initiative
WHO: World Health Organization

## References

[1] World Healh Organization. United Nations Children's Fund (UNICEF). Global Immunization data. Geneva:WHO;2014.Availablefrom:http://www.who.int/immunization/monitoring_surveillance/global_immunization_data.pdf. Accessed 13 Apr 2015.

[2] Ehreth J (2003). The global value of vaccination. Vaccine.

[3] Ministry Of Health (MOH). Republic of Senegal. EPI Comprehensive multiyear Plan 2012–2016. 2011. 76. Available from: http://www.epi.gov.pk/wp-content/uploads/2014/09/National-cMYP.pdf. Accessed 24 Jun 2015.

[4] United State Agency for International Development (USAID).Senegal: Routine immunization overview. 2013. p. 4. Available from: pdf.usaid.gov/pdf_docs/PA00JTF2.pdf. Accessed 25 June 2014.

[5] Seck I, Faye A, Mbacké Leye MM, Bathily A, Camara MD, Ndiaye PDA. Measles epidemic and response in the region of Dakar (Senegal) in 2009. Sante Publique (Paris). 2012;24(2):121–32. 22789117

[6] Rutstein SO, Rojas G. Guide to DHS statistics. Demographic and health surveys methodology. 2006. p 61. Available from: https://dhsprogram.com/pubs/pdf/DHSG1/Guide_to_DHS_Statistics_29Oct2012_DHSG1.pdf. Accessed 10 Dec 2016.

[7] Bekondi C, Zanchi R, Seck A, Garin B, Giles-vernick T, Gody JC, et al. HBV immunization and vaccine coverage among hospitalized children in Cameroon, Central African Republic and Senegal: a cross-sectional study. BMC Infect Dis. 2015:25–8. 10.1186/s12879-015-1000-2 PMC4499446 26164361

[8] Ndiaye NDM, Ndiaye P, Diédhiou A, Guèye AS (2009). Facteurs d’abandon de la vaccination des enfants ages de 10 a 23 mois a Ndoulo (Senegal). Cah d’études Rech Francoph / Santé.

[9] MEASURE: Guide to DHS statistics. Demographic and Health Surveys Methodology. 2013. Available from: http://pdf.usaid.gov/pdf_docs/pnaec362.pdf. Accessed 10 March 2015.

[10] Agence Nationale de la Statistique et de la Démographie (ANSD) [Sénégal], et ICF International. 2012. Enquête Démographique et de Santé à Indicateurs Multiples au Sénégal (EDS-MICS) 2010-2011. Retrieved from: http://dhsprogram.com/publications/publication-FR258-DHS-Final-Reports.cfm.

[11] George K, Victor SAR (1990). Reliability of mother as an informant with regard to immunisation. Indian J Pediatr.

[12] Gareaballah ETLB (1989). The accuracy of mother’s report about their children vaccination status:WHO bulletin. Bull World Health Organ.

[13] Lakew Y, Bekele A, Biadgilign S (2015). Factors influencing full immunization coverage among 12–23 months of age children in Ethiopia: Evidence from the national demographic and health survey in 2011. BMC Public Health BMC Public Health.

[14] Rainey JJ, Watkins M, Ryman TK, Sandhu P, Bo A, Banerjee K (2011). Reasons related to non-vaccination and under-vaccination of children in low and middle income countries : Findings from a systematic review of the published literature ,1999 – 2009. Vaccine.

[15] Funmilayo A. Determinants of full child immunization among 12-23 months old in Nigeria. Msc thesis. University of the Witswatersrand. Demography and Population Studies Department. 2015.

[16] Greenland S. Commentary Modeling and Variable Selection in Epidemiologic Analysis. Am J Public Health. 1989;79(3):340–9. 10.2105/ajph.79.3.340 PMC1349563 2916724

[17] Hosmer DW, Hosmer T, Le Cessie SLS. A comparison of goodness-of-fit tests for the logistic regression model. Stat Med. 16(9):965–80. 10.1002/(sici)1097-0258(19970515)16:9<965::aid-sim509>3.0.co;2-o 9160492

[18] Favin M, Steinglass R, Fields R, Banerjee K, Sawhney M (2012). Why children are not vaccinated: a review of the grey literature. Int Health Royal Society of Tropical Medicine and Hygiene.

[19] Bbaale E (2015). Factors influencing Childhood Immunization in Uganda. J Health Popul Nutr.

[20] Odusanya OO, Alufohai EF, Meurice FP, Ahonkhai VI (2008). Determinants of vaccination coverage in rural Nigeria. BMC Public Health.

[21] Delamonica E, Minujin A, Gulaid J (2005). Monitoring equity in immunization coverage. Bull World Health Organ.

[22] Donnell OO (2007). Access to health care in developing countries : breaking down demand side barriers. Cad Saude Publica.

[23] Negeri EL, Heyi WD (2015). An assessment of child immunization coverage and its determinants in Sinana District, Southeast Ethiopia. BMC Pediatr.

[24] Kibel M, Westwood Tony SH (2013). Child Health for All: A Manual for Southern Africa 5th Edition.

[25] Lynch J, Smith G, Harper S (2004). Is Income Inequality a Determinant of Population Health? Part 1. A Systematic Review. Milbank.

[26] World Healh Organization (WHO). United Nations Children's Fund (UNICEF). State of the world’s vaccines and mmunization.:WHO;2010. Availablefrom:http://www.who.int/immunization/monitoring_surveillance/globalimmunization_data.pdf. Accessed 13 Apr 2015.

